# Reporting of unintended events in an intensive care unit: comparison between staff and observer

**DOI:** 10.1186/1471-227X-5-3

**Published:** 2005-05-27

**Authors:** Maurizia Capuzzo, Imad Nawfal, Matilde Campi, Vanna Valpondi, Marco Verri, Raffaele Alvisi

**Affiliations:** 1 Department of Surgical, Anaesthetic and Radiological Sciences, Section of Anaesthesiology and Intensive Care, University Hospital of Ferrara, Corso Giovecca 203 44100 Ferrara, Italy

## Abstract

**Background:**

In order to identify relevant targets for change, it is essential to know the reliability of incident staff reporting. The aim of this study is to compare the incidence and type of unintended events (UE) reported by facilitated Intensive Care Unit (ICU) staff with those recorded concurrently by an observer.

**Methods:**

The study is a prospective data collection performed in two 4-bed multidisciplinary ICUs of a teaching hospital. The format of the UE reporting system was voluntary, facilitated and not necessarily anonymous, and used a structured form with a predetermined list of items. UEs were reported by ICU staff over a period of 4 weeks. The reporting incidence during the first fourteen days was compared with that during the second fourteen. During morning shifts in the second fourteen days, one observer in each ICU recorded any UE seen. The staff was not aware of the observers' study. The incidence of UEs reported by staff was compared with that recorded by the observers.

**Results:**

The staff reported 36 UEs in the first fourteen days and 31 in the second.. The incidence of UE detection during morning shifts was significantly higher than during afternoon or night shifts (p < 0.001). Considering only working day morning shifts, the rate of UE reporting by the staff per 100 patient days was 26.9 (CI 95% 16.9–37.0) in the first fourteen day period and 20.3 (CI 95% 10.3–30.4) in the second. The rate of UE detection by the observers was 53.1 per 100 patient days (CI 95% 40.6–65.6), significantly higher (p < 0.001) than that reported concurrently by the staff. There was excellent agreement between staff and observers about the severity of the UEs recorded (Intraclass Correlation Coefficient 0.869). The observers recorded mainly UEs involving Airway/mechanical ventilation and Patient management, and the staff Catheter/Drain/Probe and Medication errors (p = 0.025).

**Conclusion:**

UE incidence is strongly underreported by staff in comparison with observers. Also the types of UEs reported are different. Invaluable information about incidents in ICU can be obtained in a few days by observer monitoring.

## Background

Voluntary and anonymous Critical Incident Reporting (CIR) in Intensive Care Units (ICUs) is a technique for collecting information about any unintended event or outcome that reduced or could have reduced the safety margin for the patient. CIR has been used in single adult [1-5] as well as neonatal-pediatric [6] ICUs, and implemented at a national level in Australia [7-9].

It has been claimed that the rate of critical incidents could be a marker of the quality of care [5]. Unfortunately, CIR does not provide an objective numerator and there is no unequivocal denominator. Taking patient days as denominator, Bracco and co-workers [4] reported 777 critical incidents (241 of them were human errors) in 2810 patient days, giving a rate of 27.4 critical incidents (8.6 human errors) per 100 patient days, while Flaatten and Hevroy [10] found 2.7 errors per 100 patient days and, more recently, Osmon and co-workers [11] reported 8.93 medical errors per 100 ICU days. On the other hand, a pharmacist identified 187 medication administration errors in 851 patients [12], and an observer detected 132 medication errors in 88 patient days of observation [13]. Moreover, it has been shown that ICU staff reported only 61% of the errors detected by an external observer [14]. In some cases, such differences may reflect different definitions, but in others [3-6] they suggest that the real size of the patient safety problem in ICU is unknown.

The ultimate goal of incident reporting is to implement strategies to prevent recurrence [15]. Therefore, to ensure that relevant targets for change are recognized, it is essential to know the reliability of staff reporting.

The aim of this study is to compare the incidence and type of unintended events reported by facilitated ICU staff with those recorded concurrently by an observer.

## Methods

### Design

The study lasted for 28 days in 2003, from 24 November (7.00 am) to 22 December (7.00 am). Staff reporting of UEs on structured forms was active throughout the period. The UEs reported by the staff during the first fourteen days were compared with those reported during the second fourteen to detect any change in staff reporting.

During morning shifts (7.00 am-14.00 pm) in the second fourteen days (weekends and holidays were excluded), one observer in each ICU who had no other duties recorded any UE seen. The observations were performed concurrently in two ICUs and compared with the incidence of UE reporting by staff over the same period of time, to measure the reliability of spontaneous reporting.

The Ethics Committee of the Hospital approved the study.

### Setting

The study was performed in two four-bed multidisciplinary ICUs located in different structures of the same 916-bed teaching hospital. Each ICU consists of a wide room with four beds allowing direct patient observation, with sliding curtains between beds.

Both ICUs have the same nurse-to-patient ratio, which is 1:2 (excluding 1 daytime unit sister per each ICU, with organizational and administrative tasks), and three nurse shifts: morning (7.00–14.00), afternoon (14.00–22.00) and night (22.00-7.00). In the ICUs, medical care is provided by specialists in anaesthesia and intensive care according to national rules. The ICUs have one overall medical director. A full time specialist and a resident doctor manage each ICU in the daytime (8.00–20.00), while a specialist and a resident doctor cover both ICUs at night (20.00-8.00).

### Procedure

Before the study was initiated, several meetings with the research team were held to familiarize the ICU staff with the concept of CIR, to describe the non-punitive nature of the study, and to develop a structured form with a predetermined list of UEs for data collection. The form was tested in both ICUs, modified according to staff comments (excluding items never used and adding space for possible items not previously considered) and then implemented. The list of the items in the structured reporting form is given in the Table 1. The staff was instructed to report any UE irrespective of whether it was on the predetermined list, and to use the appropriate space in the form if the item was not listed.

**Table 1:** List of the items in the structured reporting form.


ICU where the UE has occurred	Shift of UE detection: Morning
Reporter's qualification: Nurse, Physician	Afternoon
Date of detection	Night
	
**Type of UE: **Problems with airway/mechanical ventilation	**Type of UE: **Problems with catheter/drain/probes
Accidental extubation	Unplanned removal
Unplanned reintubation	Dislodgement
Tracheal tube obstruction	Inappropriate opening
Tracheal cuff leakage	Inappropriate disconnection
Incorrect ventilator setting	
Ventilator auto cycling	**Type of UE: **Problems with medication
Turn off of heated humidifier	Prescription error
Turn off of ventilator alarms	Transcription error
	Wrong dose
**Type of UE: **patient management:	Wrong route of administration
Delayed treatment	
Incorrect patient positioning	**Type of UE: **unit management
Documentation lacking	Organization
Documentation reported incorrectly or inaccurately	Communication
	Equipment failure
Turn off of oxygen saturationalarm	
	**Other problem**:................................
**Severity of the unintended event**	
Intercepted by the staff	
Self resolving	
Minor	
Serious	

During the period of the study, the Director of the Unit enhanced reporting by emphasizing its relevance during ward rounds, encouraging staff to fill in the form immediately after discovering any UE, and discussing relevant (minor or serious) reports weekly with the whole ICU staff. Reporting was facilitated by the cooperative attitude of all the staff and by allowing but not requiring anonymity. The format of UE reporting system was voluntary, non-punitive, facilitated and not necessarily anonymous.

Blank forms were available on the nursing desk and completed ones were stored in a free deposit box, which was emptied every evening by those responsible for the study, who first analysed the reports. A poster with guidelines for reporting, definitions of UEs, some examples and the name of the person responsible for the research was displayed on the wall next to the forms.

During morning shifts in the second fourteen day period of the study, two residents attending the Specialist School in Anaesthesia and Intensive Care of the University (one in each ICU) acted as observers. They had already performed a one-year training period in general ICU and, before the beginning of the study, they had studied CRI methodology, analyzed precise definitions of variables in the literature and received instruction, with examples, about the definitions of UEs used in the present study. The observers, like the staff, were instructed to report any UE irrespective of whether it was in the predefined list, and to use the appropriate space in the form if the item was not listed. The observers used the same rules and structured forms as the staff, but they filled in a new form for each UE in a separate room and retained it in a separate box until the end of the study. The staff was not aware of the observers' study; the presence of the observers was explained by the collection of data for a different study.

### Definitions

For the purpose of the study, any unintended event (UE) that reduced or could have reduced the safety margin for the patient while in ICU was considered. UEs occurring during transport or in other areas of the hospital were not considered. The following information, collected for each UE (items listed in the Table 1), was analyzed: date and shift of detection, reporter's qualification (nurse or physician), type and severity. The type of UE was categorized as follows [8]: Airway/mechanical ventilation, Catheter/Drain/Probe, Medication error, Patient management, Unit management, and Other (with space for details). UE severity was classified according to the reporter's opinion of the outcome and defined as follows:

• intercepted and spontaneously rectified by the staff before the patient could be affected (for example: a doctor prescribed a wrong dose of a drug, the nurse realized that it was a mistake and informed the doctor, who corrected the error so that the patient received the correct dose);

• self-resolving without specific treatment at the time of the event without harm to the patient (for example: accidental removal of a nasogastric tube that was no longer necessary, or alarms turned off after the end of nursing activities or physician round);

• minor at the time of the event but requiring transient increase in surveillance or adjustment of treatment (for example: unplanned intubation after planned extubation, without major physiological complications);

• serious, i.e. life-threatening or responsible for an increased length of hospitalization.

### Data analysis

Statistical analysis was carried out using a software package (SigmaStat 2.0) and p values less than 0.05 were considered significant. Proportions were reported with 95% Confidence Intervals (CI) [16] and compared by a z test.

For the purpose of the study, the 4-week period was split into two intervals of fourteen days: the first (A) included 10 working and 4 non-working (weekend) days, and the second (B) included 9 working and 5 non-working days (4 weekend days and 1 national holiday). The following comparisons were performed: 1) between UEs reported by the staff during periods A and B, to exclude any effect of the presence of the observer; 2) between number and type of UEs reported by staff and observers during period B, to measure the reliability of spontaneous reporting; 3) between the severity scores given by staff and observer to the same UE, using an Intraclass Correlation Coefficient (ICC). The ICC assesses agreement, ranging from 0 (no agreement) to 1 (scores identical). Values higher than 0.80 indicate good agreement.

## Results

### Patient characteristics

During the period of study 31 patients were admitted to or already present in the ICU. The median age was 68 y (range 28–92), median duration of mechanical ventilation was 3 days (range 0–21), and median length of stay in ICU was 6 days (range 1–43). The median values of SAPS II [17] and APACHE II [18] were 37 (range 7–67) and 15 (range 7–31) respectively. One patient died in the ICU and 10 patients died in hospital. The characteristics of the ICU patients present during periods A and B are shown in table 2.

**Table 2:** Patients present in the ICU during the first and second 14 day periods.

Period of fourteen days	first	second
Number of patients present in the period	18	20
Age (years): median	74	73
Type of ICU admission		
Surgical planned	6	8
Surgical unplanned	7	7
Medical	5	5
SAPS II at ICU admission: median	40	39
APACHE II at ICU admission: median	18	15
Number of ICU days (hours/24) in the study	110	99
Number of ventilation days (hours/24) in the study	71	59
Available ICU bed days	112	112

The total number of patients surveyed was 31. Seven patients (median values of age 76 y, SAPS II 47, APACHE II 19) were present in both periods. Numbers of ICU and ventilation days are given as exact numbers: counted hours divided by 24.

### Bed day occupation

The exact number of bed days occupied (patient days), computed as sum of the hours spent by each patient in the ICU during the period of study divided by 24, was 209 over the whole 4 week period. The numbers of patient days during the working day morning shifts of periods A and B were 78 and 64, respectively; the numbers of bed days available were 80 and 72, respectively; and bed occupancies were 98% and 89%, respectively.

### Unintended events reported by the staff

The staff reported 36 UEs in period A and 31 in period B. There was no significant difference in incidence (33 vs 31/100 patient days) between the two periods (p = 0.872), or between the number of UEs reported in periods A and B by physicians and nurses (31 and 25 versus 5 and 6, respectively; p = 0.786). Of the 67 UEs reported by the staff, 52 occurred during working and 15 in non-working days, with incidences of 36 and 22/100 patient days, respectively (p = 0.061). Considering the 4 weeks as a whole, the incidence of UEs detected during morning shifts (22/100 patient days) was significantly higher(p < 0.001) than in afternoon (6/100 patient days) or night (4/100 patient days) shifts.

Of the 67 UEs reported by staff, 8 were classified as intercepted by the staff (for instance, 5 medication errors in drug prescription were rectified before administration), 45 as self-resolving, 13 as minor and 1 as serious. The last was a ventricular fibrillation, treated without sequelae. The patient's serum potassium level was low according to a laboratory test on a blood sample taken in the morning. Potassium chloride 40 mEq was prescribed to be added to the parenteral bag. The nurse injected the drug into the bag without stopping the infusion pump. In 1 min, the patient, who was intubated but alert, became agitated, so the physician and the nurse hastened to him. Afterwards, the patient lost consciousness with the EGC monitor showing ventricular fibrillation; defibrillation was performed in less than 30 s.

### Unintended events detected by observer

In the working day morning shifts of period B, observers detected 34 UEs in 64 patient days. These UEs were classified as intercepted by the staff (3 cases), self-resolving (25), and minor (6). The types of UEs detected by observers were as follows: 13 were in the Airway/mechanical ventilation category, 3 Catheter/Drain/Probe, 5 Medication errors, 12 Patient management and 1 Unit management.

### Comparison between staff and observer reports

In the working day morning shifts of period A, the rate of UEs per 100 patient days was 26.9 (21/78), with CI 95% = 16.9–37.0. In the working day morning shifts of period B, the observers detected 21 UEs not recorded by the staff. No UE was reported by staff and missed by observers. The rate of UEs per 100 patient days was 20.3 (CI 95% = 10.3–30.4) according to the staff and 53.1 (CI 95% = 40.6–65.6) according to the observers (p < 0.001). Of the UEs detected by the ICU observers, the staff reported 37.5% in one ICU and 38.5% in the other.

The staff and the observer recorded the same severity in 11 of the 13 UEs that were recorded by both (1 intercepted by the staff, 7 self resolving and 3 minor); of the remainder, the observer scored one UE higher than the staff (minor vs self-resolving) and the other lower (self-resolving vs minor). The ICC for the staff and observer severity ratings of the 13 UEs was 0.869, showing excellent agreement.

The types of UEs reported by the staff during the whole study period (4 weeks), and those reported during period B (morning shifts in working days) by the staff and the observers, are shown in table 3. Of the UEs reported by observers, 6 out of the 13 Airway/mechanical ventilation events involved turning off ventilator alarms and 5 out of the 12 patient management events involved saturation alarms being switched off. The incidences of the 5 types of UE recorded by the observers were significantly different (p = 0.025) from those recorded by staff over the whole study period. No difference was found in the incidences of the 5 types of UE reported by staff during the 4-week study period or in the working day morning shifts of period B (p = 0.265).

**Table 3:** Types of unintended events according to the time of reporting and reporters.

Period of fourteen days	all	first	second
Days	all	working	working
Shifts	all	morning	morning
Reporter	staff	staff	observer
N. of events concerning			
Airway/mechanical ventilation	13 (19%)	2 (15%)	13 (38%)
Catheter/Drain/Probe	18 (27%)	1 (8%)	3 (9%)
Medication errors	19 (28%)	3 (23%)	5 (15%)
Patient management	13 (19%)	6 (46%)	12 (35%)
Unit management	4 (6%)	1 (8%)	1 (3%)
Total number of events	67	13	34

Statistical significance: staff (entire period, first column) vs staff (first fourteen days, second column) p = 0.265 (chi square 5.222); staff (entire period, first column) vs observers (last column) p = 0.025 (chi square 11.127).

## Discussion

This study demonstrates that the rate of spontaneous reporting of UEs by ICU staff is less than half an observer's reporting rate. Therefore, information obtained by CIR markedly underestimates the real UE frequency. Even more important, the type of UEs spontaneously reported by staff is different from that recorded by an observer using the same criteria as the staff. If we had had to prioritize changes on the basis of staff reporting in our setting (Table 3, first column), we would have devoted more attention to the Catheter/Drain/Probe and Medication error categories. In contrast, Airway/mechanical ventilation and Patient management were the most frequent types of UE recorded by observers. Therefore, spontaneous staff reporting does not appear reliable enough to mirror the truth.

Generally, 12% (8 of 67) of UEs reported by the staff and 9% (3 of 34) of those detected by observers were intercepted by the staff, suggesting a measure of the efficiency of control in our setting. Most of the UEs recorded by observers involved latent errors in alarm settings, i.e. potential problems within the system [19]. Surprisingly, staff members were not aware of this kind of problem and only the observer reports allowed us to identify it.

In this study, most UEs (83%) were reported by physicians. This could be due to the facilitated format of reporting, i.e. the enhancement of immediate reporting by the attending physicians, with stronger emphasis on the report than on the anonymity; and to the cooperative attitude of all the staff, which allowed physicians to record on the form an event discovered by a nurse. The lack of double reports indicates that nurses and doctors, attending and resident, were informed about UEs already reported; the report forms remained in an open box throughout the day of the event. On the other hand, nurses reported 43% of critical incidents [3] and 59% of medical errors [11]. Moreover, nurses reported 74% of incidents in the Australian Incident Monitoring Study published in 1996 [7] and 49% of the incidents collected in 2003, by facilitated incident monitoring, in an ICU where CIR had been used for more than 5 years [5]. This finding and our results suggest that physician reporting increases when it is facilitated and, possibly, not anonymous.

The fact that the highest frequency of UEs was discovered in morning shifts agrees with the findings of Donchin and coworkers [14], who studied the diurnal distribution of errors, and Frey and coworkers, who analyzed critical incidents in pediatrics [6]. Other authors found the highest incidence of critical incidents [3] or errors [10] in afternoon shifts. Our finding is consistent with the high number of activities performed, especially by physicians, during the morning. Nevertheless, in the present study, the time of UE reporting was taken as the time of UE detection, and we cannot exclude the possibility that some UEs reported in morning shifts had actually occurred during other shifts.

The study has some limitations. The use of a predefined list of items could have induced both staff and observers to pay attention mainly to the listed items, even though space was allowed for additional ones. Therefore, we can not exclude the possibility that other UEs were missed. Nor can we exclude the possibility that the staff reported fewer events in period B than in period A due to a lessening of the initial enthusiasm, although there was no statistically significant difference. However, the lower number of patient days in working day morning shifts during period B (64 vs 78 in period A) could explain the lower number of events reported in terms of both direct (reduced number of patients at risk, fewer procedures) and indirect (reduced nursing workload) effects. Another limitation is that nursing workload during the study period was not measured. We did not investigate inter-observer reliability, but agreement in responses to questions concerning adverse event reporting has been shown to be good [20]. Indeed, the staff of the two ICUs reported similar percentages of the events recorded by the observers. This finding supports our general conclusion that the incidence of unintended events is markedly underestimated by spontaneous reporting. Finally, no attempt was made to investigate whether the UEs reported were preventable, considering that it has already been shown that most incidents are preventable [3,5].

## Conclusion

Our study demonstrates that the incidence of UEs is markedly underestimated by spontaneous staff reporting in comparison with observers' recording. Even more important, the type of UE reported is different. One final implication of this study, performed in a single centre and over a short period of time, is of general value: invaluable information about incidents in ICU can be obtained in a few days by observer monitoring.

## List of abbreviations used

Intensive Care Units ICU

unintended events UE

Critical Incident Reporting CIR

Simplified acute physiology score SAPS II

Acute Physiology And Chronic Health Evaluation APACHE II

## Competing interests

The author(s) declare that they have no competing interests.

## Authors' contributions

MC conceived and proposed the study, participated in the design and in meetings to prepare the staff, performed the statistical analysis and draft the manuscript. IM and MC participated in the designing and preparing the structured form, collected data as observers, participated in the analysis and in drafting the article. VV and MV participated in meetings with the staff to prepare the structured form, stimulated the staff to reporting, discussed the reports with the staff and participated in drafting the article. RA (Director of the Unit) participated in the design, enhanced reporting at round visits, and critically revised the article. At the time of the study, MC, IM, MC and RA were completely aware of the study (comparison between staff and observers), while VV and MV were informed only about the staff data reporting.

All authors read and approved the final manuscript.

## Pre-publication history

The pre-publication history for this paper can be accessed here:


